# Efficacy and safety of active negative pressure peritoneal therapy for reducing the systemic inflammatory response after damage control laparotomy (the Intra-peritoneal Vacuum Trial): study protocol for a randomized controlled trial

**DOI:** 10.1186/1745-6215-14-141

**Published:** 2013-05-16

**Authors:** Derek J Roberts, Craig N Jenne, Chad G Ball, Corina Tiruta, Caroline Léger, Zhengwen Xiao, Peter D Faris, Paul B McBeth, Christopher J Doig, Christine R Skinner, Stacy G Ruddell, Paul Kubes, Andrew W Kirkpatrick

**Affiliations:** 1 Department of Surgery, University of Calgary and the Foothills Medical Centre, North Tower 10th Floor, 1403-29th Street Northwest, Calgary, Alberta, T2N 2T9, Canada; 2 Department of Community Health Sciences (Divisions of Epidemiology and Biostatistics), University of Calgary, TRW (Teaching, Research, and Wellness) Building, 3rd Floor, 3280 Hospital Drive Northwest, Calgary, Alberta, T2N 4Z6, Canada; 3 Department of Critical Care Medicine, University of Calgary and the Foothills Medical Centre, Ground Floor McCaig Tower, 3134 Hospital Drive Northwest, Calgary, Alberta, T2N 5A1, Canada; 4 Department of Oncology, University of Calgary and the Foothills Medical Centre, 1403-29th Street Northwest, Calgary, Alberta, T2N 2T9, Canada; 5 Regional Trauma Program, University of Calgary and the Foothills Medical Centre, 1403-29th Street Northwest, Calgary, Alberta, T2N 2T9, Canada; 6 Alberta Health Services – Research Excellence Support Team, University of Calgary and the Foothills Medical Centre, South Tower, 1403-29th Street Northwest, Calgary, Alberta, T2N 2T9, Canada; 7 Hotchkiss Brain Institute, University of Calgary and the Foothills Medical Centre, Health Research Innovation Centre, 3330 Hospital Drive Northwest, Calgary, Alberta, T2N 4N1, Canada; 8 Calvin, Phoebe and Joan Snyder Institute for Chronic Diseases, University of Calgary and the Foothills Medical Centre, Health Research Innovation Centre, 3280 Hospital Drive Northwest, Calgary, Alberta, T2N 4N1, Canada; 9 Division of Critical Care Medicine, Department of Medicine, University of British Columbia and St. Paul’s Hospital, 1081 Burrard Street, Vancouver, British Columbia, V6Z 1Y6, Canada

**Keywords:** Abdominal injuries, Damage control laparotomy, Inflammation, Negative pressure wound therapy, Randomized controlled trial, Sepsis, Temporary abdominal closure, Wounds and injuries

## Abstract

**Background:**

Damage control laparotomy, or abbreviated initial laparotomy followed by temporary abdominal closure (TAC), intensive care unit resuscitation, and planned re-laparotomy, is frequently used to manage intra-abdominal bleeding and contamination among critically ill or injured adults. Animal data suggest that TAC techniques that employ negative pressure to the peritoneal cavity may reduce the systemic inflammatory response and associated organ injury. The primary objective of this study is to determine if use of a TAC dressing that affords active negative pressure peritoneal therapy, the ABThera Open Abdomen Negative Pressure Therapy System, reduces the extent of the systemic inflammatory response after damage control laparotomy for intra-abdominal sepsis or injury as compared to a commonly used TAC method that provides potentially less efficient peritoneal negative pressure, the Barker’s vacuum pack.

**Methods/Design:**

The Intra-peritoneal Vacuum Trial will be a single-center, randomized controlled trial. Adults will be intraoperatively allocated to TAC with either the ABThera or Barker’s vacuum pack after the decision has been made by the attending surgeon to perform a damage control laparotomy. The study will use variable block size randomization. On study days 1, 2, 3, 7, and 28, blood will be collected. Whenever possible, peritoneal fluid will also be collected at these time points from the patient’s abdomen or TAC device. Luminex technology will be used to quantify the concentrations of 65 mediators relevant to the inflammatory response in peritoneal fluid and plasma. The primary endpoint is the difference in the plasma concentration of the pro-inflammatory cytokine IL-6 at 24 and 48 h after TAC dressing application. Secondary endpoints include the differential effects of these dressings on the systemic concentration of other pro-inflammatory cytokines, collective peritoneal and systemic inflammatory mediator profiles, postoperative fluid balance, intra-abdominal pressure, and several patient-important outcomes, including organ dysfunction measures and mortality.

**Discussion:**

Results from this study will improve understanding of the effect of active negative pressure peritoneal therapy after damage control laparotomy on the inflammatory response. It will also gather necessary pilot information needed to inform design of a multicenter trial comparing clinical outcomes among patients randomized to TAC with the ABThera *versus* Barker’s vacuum pack.

**Trial registration:**

ClinicalTrials.gov identifier http://www.clicaltrials.gov/ct2/show/NCT01355094

## Background

Intra-abdominal sepsis and abdominal trauma constitute major international public health problems [1]. As limited treatments aside from source control and antimicrobial therapy exist for severe intra-abdominal infections, intra-peritoneal sepsis is associated with significant morbidity and mortality [2]. Moreover, trauma affects approximately 700 million people, including 30 million North Americans and 2 million Canadians, worldwide each year [1,3]. These injuries result in 5 million deaths, with blunt and penetrating abdominal trauma constituting a substantial proportion of trauma-related mortality [1,3,4].

Deranged patient physiology as a result of massive hemorrhage and/or contamination from hollow viscus or pancreaticobiliary injuries is an important contributor to poor outcomes after intra-abdominal sepsis or injury [5,6]. In an attempt to limit this deranged physiology, damage control laparotomy, or abbreviated initial laparotomy followed by temporary abdominal closure (TAC) and planned re-operation after intensive care unit (ICU) resuscitation, is increasingly used to manage intra-abdominal bleeding and contamination among these patients [5-10]. The stages of damage control laparotomy broadly include: (1) limited initial operation with temporary control of hemorrhage and contamination; (2) application of a TAC device; (3) ICU resuscitation; and (4) re-operation with attempted completion of definitive surgical repairs after normalization of patient physiology [5,7-9].

A common component of the deranged physiologic response after intra-abdominal sepsis or injury is inflammation. Although intra-abdominal sepsis or injury also results in a systematic inflammatory response, evidence suggests that this response is exaggerated in the peritoneum [11,12]. Microcirculatory disruption after septic or hemorrhagic shock may lead to loss of intestinal barrier function, bowel edema, and formation of pro-inflammatory-mediator-rich ascites [13-18]. This inflammatory ascites may serve as a motor for sepsis, which may perpetuate systemic inflammation and result in multi-organ dysfunction syndrome [11,12,14,18]. In support of this, the levels of select peritoneal cytokines have been reported to be significantly different between animals that survived as compared to those who died in animal models of intra-abdominal sepsis [19]. However, although a number of mediators have been associated with sepsis or injury, relatively little is known about their temporal peritoneal and systemic expression after intra-abdominal sepsis or injury, including abdominal surgery (Table 1) [16,20-43].

**Table 1:** Inflammatory mediators associated with intra-abdominal sepsis or injury, including abdominal surgery, among studies of animals or humans

**Mediator**	**Potential role in intra-abdominal sepsis or injury**	**Reference(s)**
CRP	A serum marker of sepsis that increases in concentration in plasma following abdominal surgery	[20-22]
Haptoglobulin	Elevated expression in blood leukocytes following severe blunt trauma	[23]
IL-1ra	Elevated in plasma following trauma; elevated expression in blood leukocytes following severe blunt trauma	[23,24]
IL-6	Potent inflammatory mediator and marker of sepsis; elevated levels correlate with length of hospital stay, complications, and mortality among patients with intra-abdominal sepsis; elevated levels in peritoneal fluid in a porcine model of intra-abdominal sepsis; elevated plasma/serum levels following abdominal surgery; elevated plasma levels following severe trauma associate with injury severity, development of organ dysfunction, and poor outcomes, including mortality	[21-23,25-28,44-50]
IL-8	Potent neutrophil chemoattractant; elevated expression in blood leukocytes following severe blunt trauma	[23,29]
IL-10	Elevated serum levels during intra-abdominal sepsis; blocks pro-inflammatory cytokine release; elevated after abdominal surgery	[30-32]
IL-15	Elevated levels in serum correlate with organ dysfunction and poor patient prognosis	[33]
IL-17	Potent pro-inflammatory mediator; promotes neutrophil recruitment to the peritoneal cavity and enhanced bacterial clearance in a mouse model of intra-abdominal sepsis; elevated plasma levels in select patients following severe trauma	[28,34,35]
IL-22	Elevated serum levels during intra-abdominal sepsis	[30]
IL-33	Mediates neutrophil recruitment to peritoneum; promotes bacterial clearance and reduces mortality in a mouse model of intra-abdominal sepsis	[36]
MCP-1 (CCL2)	Potent monocyte chemoattractant; serum levels elevated in a rat model of intra-abdominal sepsis; elevated expression in blood leukocytes following severe blunt trauma	[23,37]
M-CSF	Elevated in plasma following trauma	[24]
MIF	Present early in sepsis and remains elevated for a prolonged time period; significantly higher levels in non-survivors of sepsis compared to survivors; MIF neutralization reduces mortality in a mouse model of intra-abdominal sepsis	[38,39]
PDGF	Elevated in plasma following trauma	[24]
Procalcitonin	Marker of infectious complications following abdominal surgery and negatively associated with survival; elevated after abdominal surgery	[32,40]
TNF-α	Serum levels elevated in a rat model of intra-abdominal sepsis	[37]
tPA	Enhances bacterial clearance, reduces cellular influx, increases plasma and peritoneal IL-12 and IL-10 levels, and reduces lung and liver damage in a mouse model of intra-abdominal sepsis	[16]
TRAIL	Promotes inflammatory cell recruitment to the peritoneum, enhances bacterial clearance, and reduces mortality in a mouse model of intra-abdominal sepsis; modulates apoptosis	[41,42]

CCL2, Chemokine (C-C motif) ligand 2; CRP, C-reactive protein; IL-1ra, Interleukin-1 receptor antagonist; IL-6, Interleukin-6; IL-8, Interleukin-8; IL-10, Interleukin-10; IL-15, Interleukin-15; IL-17, Interleukin-17; IL-22, Interleukin-22; IL-33, Interleukin-33; MCP-1, Monocyte chemoattractant protein-1; M-CSF, Macrophage colony-stimulating factor; MIF, Macrophage migration inhibitory factor; PDGF, Platelet-derived growth factor; TNF-α, Tumor necrosis factor-alpha; tPA, Tissue plasminogen activator; TRAIL, Tumor necrosis factor-related apoptosis-inducing ligand.

Importantly, animal data suggest that TAC techniques that employ constant negative pressure to the peritoneal cavity may remove inflammatory ascites, reduce the systemic inflammatory response, and improve organ injury and potentially outcomes [10,18]. One preclinical study randomly allocated animals with intra-abdominal sepsis to TAC with vacuum-assisted drainage of the peritoneal cavity after laparotomy and observed significantly reduced levels of the pro-inflammatory cytokines tumor necrosis factor-α (TNF-α) and interleukin (IL)-1β, -6, and −12 as compared to those who received a TAC that afforded only passive peritoneal drainage [18]. Animals with septic open abdominal wounds treated with negative pressure peritoneal therapy also demonstrated significantly reduced intestinal edema and improved pulmonary, renal, and hepatic histological features; intra-abdominal pressure (IAP); cardiac and renal function; and pulmonary compliance, with a trend towards a lower incidence of acute lung injury [18]. These animal data are interesting as several studies have reported that the pro-inflammatory cytokine IL-6 is associated with an increased risk of organ dysfunction, adverse complications, and/or mortality among trauma victims and patients with sepsis [44-50].

However, very little relevant clinical data yet exist to support that negative pressure peritoneal therapy may improve outcomes among trauma and acute care surgery patients after damage control laparotomy [10]. One systematic review and meta-analysis of largely uncontrolled case series of damage control laparotomy suggested that vacuum-assisted TAC techniques may be associated with improved outcomes among critically ill adults with contaminated open abdominal wounds [51]. However, a systematic review conducted by our group in 2012 found only 11 comparative studies, including 2 randomized controlled trials (RCTs) and 9 cohort studies, examining the efficacy and safety of negative pressure peritoneal therapy *versus* alternate TAC methods among critically ill or injured adults [10]. As only one RCT compared negative pressure peritoneal therapy with an alternate type of TAC technique [52], and all studies were associated with at least a moderate risk of bias and significant clinical heterogeneity, we concluded that there was insufficient evidence to support the preferential use of negative pressure peritoneal therapy after damage control laparotomy [10].

The primary objective of this study is to determine if use of a TAC dressing that may afford active negative pressure peritoneal therapy, the ABThera™ Open Abdomen Negative Pressure Therapy System (Kinetic Concepts Inc. (KCI), San Antonio, TX, USA) [10,53], reduces the extent of the systemic inflammatory response after damage control laparotomy for intra-abdominal sepsis or injury as compared to a commonly used TAC method that provides potentially less efficient peritoneal negative pressure, the Barker’s vacuum pack [54-56].

## Methods/Design

### Overview

**Figure 1:**
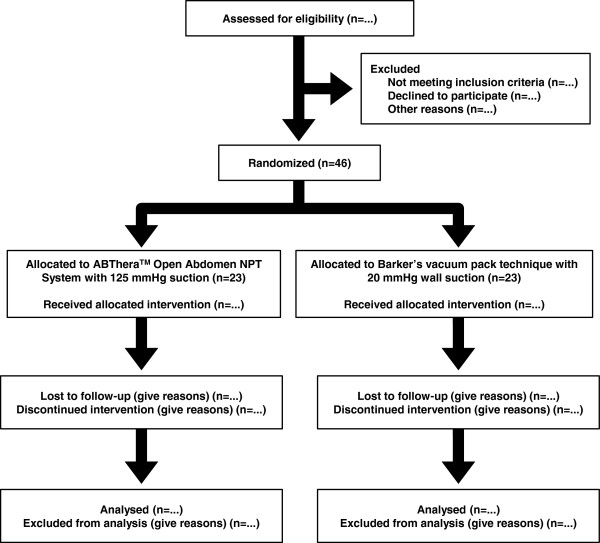
**Flow of participants in the Intra-peritoneal Vacuum Trial.** Diagram constructed according to the CONSORT statement [57].

**Figure 2:**
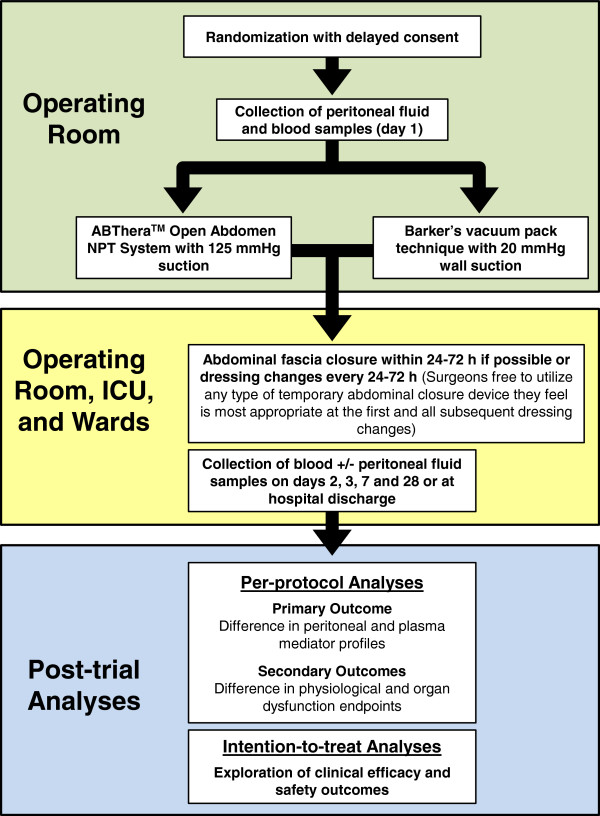
**Overview of the design of the Intra-peritoneal Vacuum Trial.** Where per-protocol treatment will be defined as the allocated temporary abdominal closure dressing having been in place for at least 24 h. ICU, intensive care unit; IL-6, interleukin-6; h, hours; NPT, negative pressure therapy.

The Intra-peritoneal Vacuum Trial is a single-center RCT, which will intraoperatively allocate critically ill or injured adults to TAC with either the ABThera™ or Barker’s vacuum pack after damage control laparotomy for intra-abdominal sepsis or injury. Although the trial in itself may not be sufficient to establish superiority of the ABThera™ over the Barker’s vacuum pack, we hypothesize superiority, and will examine for evidence of this in hypothesis tests. The CONSORT flow diagram [57] and an overview of the study design are presented in Figures 1 and 2, respectively.

### Intervention and comparator choice rationale

The ABThera™ was chosen as the intervention as its design may allow for active, and potentially equally distributed vacuum pressures throughout the peritoneal cavity and across the viscera [58], theoretically enabling effective removal of pro-inflammatory-mediator-rich intra-peritoneal fluid. The Barker’s vacuum pack was chosen as the comparator as it is a commonly used TAC technique, which has been recommended by the Eastern Association for the Surgery of Trauma as the current standard by which to measure other devices [59].

### Setting

The trial will be set at the Foothills Medical Centre (FMC) in Calgary, Alberta, Canada. The FMC is a University of Calgary-affiliated, adult, tertiary care, level one regional trauma center that affords trauma and emergency general surgical services for southern Alberta, southwest British Columbia, and southeast Saskatchewan. Postoperative critically ill or injured adults at the FMC are cared for in a 30-bed, closed, medical/surgical/neurosurgical ICU staffed by fellowship-trained intensivists.

### Patient recruitment

Recruitment into the trial will occur in the operating room once the decision has been made by the attending trauma or general surgeon to perform a damage control laparotomy and TAC. We will define an open abdomen as that requiring a TAC due to the abdominal skin and fascia not being closed after laparotomy.

Although no objective, evidence-based indications for damage control laparotomy or open abdominal management exist [60,61], the Eastern Association for the Surgery of Trauma suggest that this technique may be considered in cases of severe abdominal trauma involving hepatic, non-hepatic, or vascular injuries with intra-abdominal packing; among trauma patients with acidosis (pH ≤7.2), hypothermia (temperature ≤35°C), and clinical coagulopathy or if the patient is receiving massive transfusion; and in patients with severe intra-abdominal infection/peritonitis or necrotizing pancreatitis [59]. Other indications for the procedure suggested in 2005 by a multidisciplinary expert advisory panel on management of the open abdomen included bowel edema and a significant risk of developing abdominal compartment syndrome [62].

### Inclusion criteria

•Age ≥18 years

•Decision made by the attending trauma or general surgeon to perform a damage control laparotomy with application of a TAC device during the index laparotomy

•Requirement for postoperative ICU admission

### Exclusion criteria

•Age <18 years

•Decision made to perform a definitive or single-stage laparotomy with closure of the abdominal fascia during the index laparotomy

•Administration of intraperitoneal chemotherapy for treatment of intra-abdominal malignancies

•Pregnancy

### Eligible, non-randomized patients

Dedicated research coordinators will screen all ICU admissions daily and identify and record patients admitted with an open abdominal wound. When potentially eligible patients are identified that were not recruited, the principal investigator, a trauma and acute care surgeon (AWK), will be informed such that he can ascertain and record reason(s) why these patients were not randomized. These reasons and the list of eligible, non-randomized patients will be used to populate the CONSORT flow diagram and estimate trial feasibility (see below).

### Patient randomization

To preserve allocation concealment, randomization will be performed using a random treatment generator hosted on a dedicated, publicly available trial website (http://www.peritonealvac.com/). When an eligible patient is identified by the attending surgeon, this website will be accessed by a member of the operating room team and the patient’s last name and hospital number will be entered. The random treatment allocation will then be announced such that the attending surgeon can perform the indicated method of TAC. Variable block size randomization will be performed to ensure equal numbers of patients in each treatment group. Day 1 is considered the day of randomization.

### Consent procedures

We were granted a waiver of immediate consent for patient enrollment in the trial because: (1) all of the eligible patients will be intubated, critically ill or injured adults under general anesthesia at the time the decision to perform a damage control laparotomy and TAC is made; (2) it will be impractical for the attending surgeon or investigators to contact surrogate decision-makers as the decision to perform open abdominal management is frequently made intraoperatively, and many trauma patients arrive unidentified (as an ‘unknown male/female’); (3) the TAC dressing must be placed emergently after the decision is made to leave the abdomen open [59]; (4) the ABThera™ and Barker’s vacuum pack technique are commonly utilized for TAC at the FMC and across North America [10]; and (5) a recent systematic review by our group found insufficient evidence to suggest that the ABThera™ was superior to the vacuum pack for TAC among critically ill or injured adults requiring damage control laparotomy [10]. Trained surgical investigators will therefore approach recruited patients after their recovery to obtain delayed, written, informed consent to utilize their data for the study. These investigators will provide a written information sheet and explain the study procedures and that continued enrollment in the trial is voluntary. One copy of the consent form will be given to the patient, one will be kept in their chart, and one will be filed as trial documentation.

### Study interventions overview

The first application of the ABThera™ or Barker’s vacuum pack will be performed in the operating room by the attending trauma or general surgeon while the patient is under general anesthesia.

#### ABThera™ application

**Figure 3:**
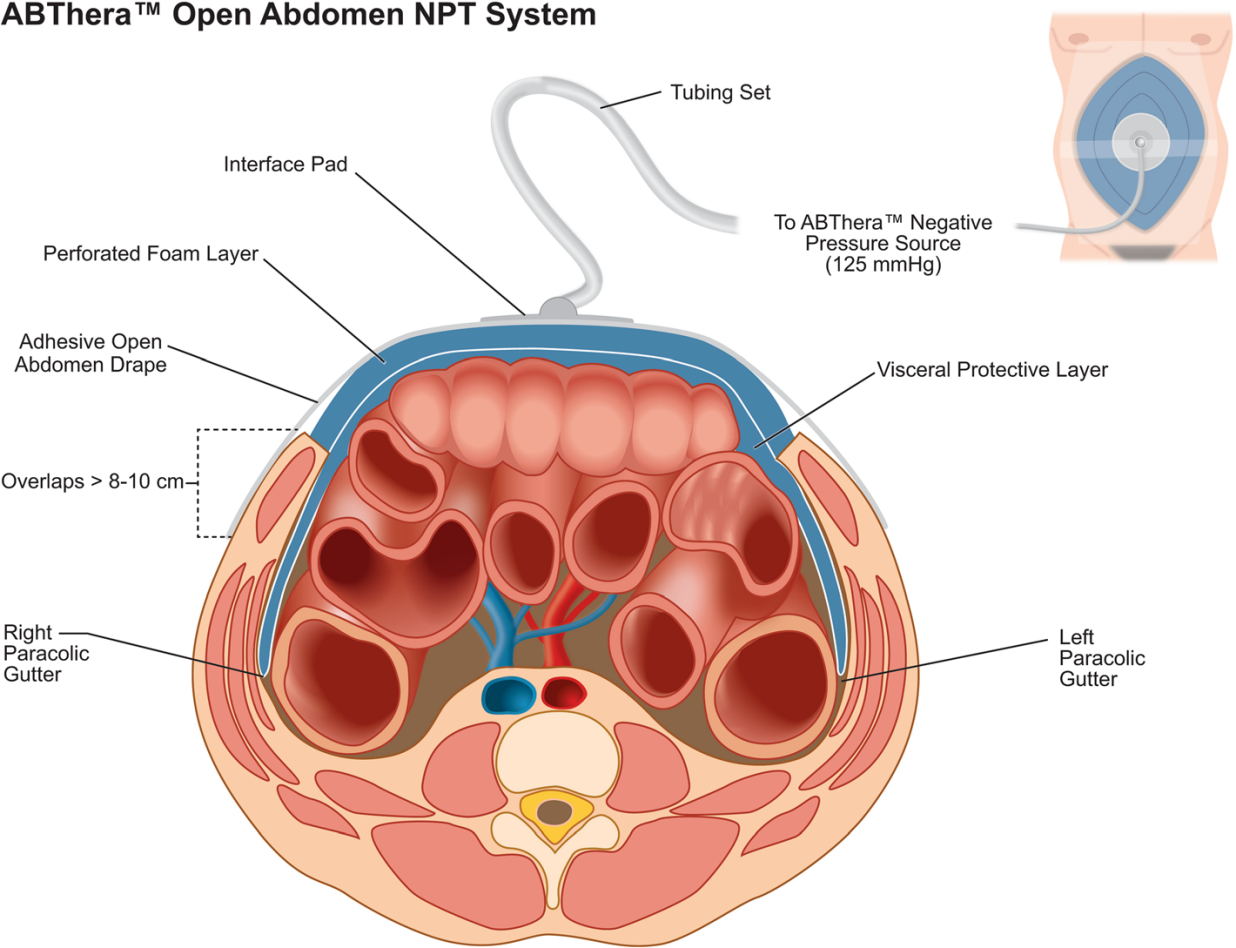
**The ABThera™ Open Abdomen NPT System.** NPT, Negative pressure therapy.

The ABThera™ (Figure 3) will be applied according to manufacturer’s recommendations [53]. The visceral protective layer will first be folded or cut to size to ensure full coverage of all viscera. It will then be gently inserted into the abdominal cavity, beneath the peritoneal lining of the abdominal wall, ensuring to work the dressing down evenly into both pericolic gutters. The more superficial perforated foam layer will then be torn to size and inserted into the open abdominal wound directly over the visceral protective layer. The adjacent skin will subsequently be sponge-dried, after which the open abdomen drape will be placed adhesive-side down onto the foam and intact skin, covering at least an 8- to 10-cm border of periwound tissue. A 2.5-cm hole will then be cut through the drape, after which the interface pad will be applied. The tubing set will subsequently be connected between the interface pad and the canister located in the negative pressure source. Finally, active negative pressure peritoneal therapy will be initiated at 125 mmHg of negative suction.

#### Barker’s vacuum pack application

**Figure 4:**
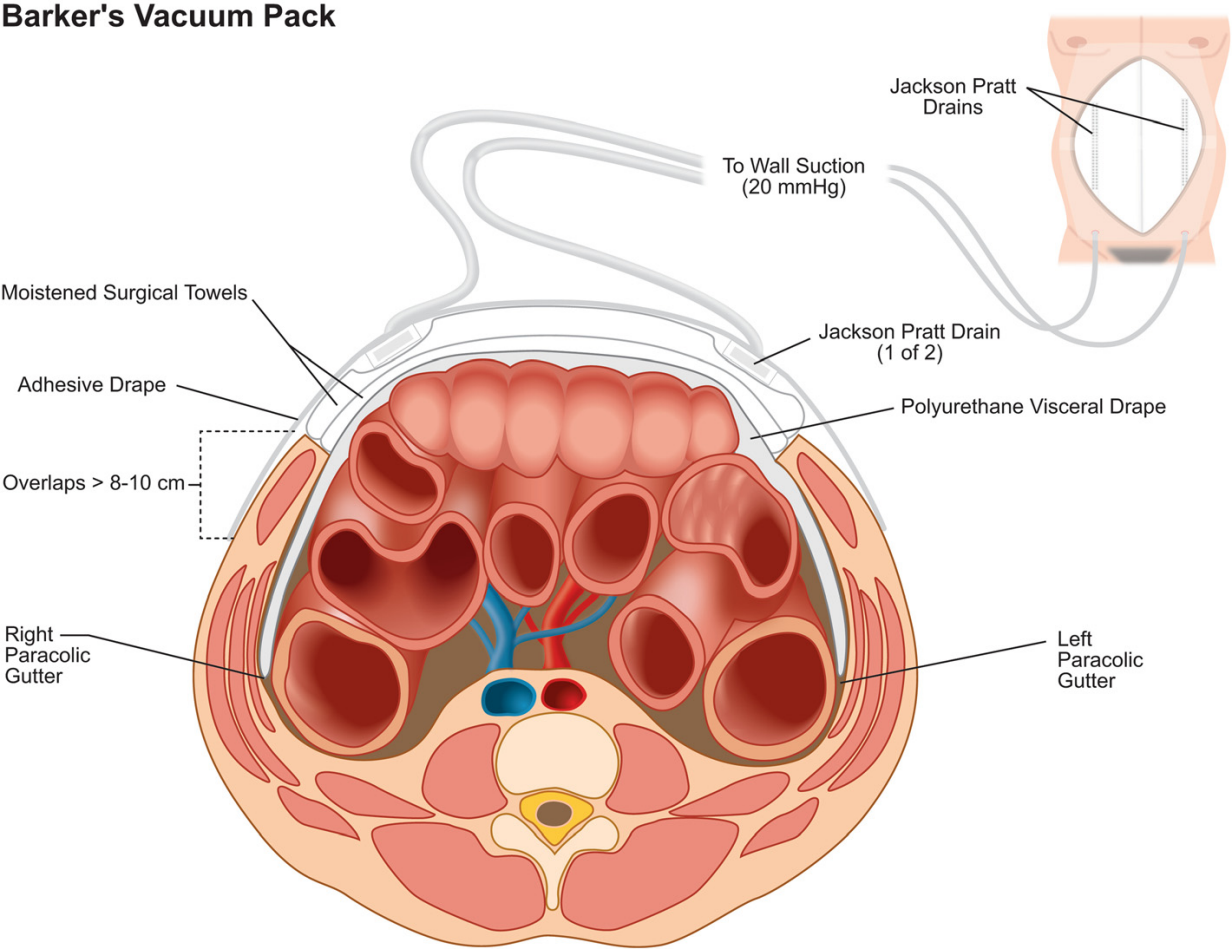
The Barker’s vacuum pack.

The Barker’s vacuum pack (Figure 4) will be applied according to our local institutional guideline and previously published descriptions [54-56]. At the completion of abdominal operation, a polyurethane sheet will be opened and may be perforated several times with a scalpel or surgical scissors. This sheet will then be placed evenly over the viscera, beneath the peritoneal cavity of the abdominal wall, extending deep into the pericolic gutters. Moistened surgical towels will subsequently be placed over the polyurethane sheet. Two closed-suction, 10-French, flat, silicone, Jackson-Pratt drains will then be placed over the moistened surgical towels. Thereafter, their drainage tubing will be tunneled approximately 3 to 5 cm below the skin in order to exit away from the laparotomy wound. The adjacent skin will subsequently be sponge-dried and covered by a large Opsite™ (Smith & Nephew Inc., St. Petersburg, FL, USA) adhesive film dressing. Finally, the drainage tubing from the two Jackson-Pratt drains will be connected to closed drain bulb suction. The Jackson-Pratt drainage tubing will be removed from these drainage bulbs after the patient arrives in the ICU, and connected to 20 mmHg of negative wall suction.

#### Reoperation and TAC dressing changes

Although the allocated TAC dressings will be left in place for a duration determined by the attending surgeon, local FMC and international guidelines suggest re-operation with attempts at abdominal fascia closure between 24 and 72 h after initial application [53,59,62]. If abdominal fascial closure is not safe or possible at this time, then a TAC dressing change is recommended [53,62]. Surgeons will be free to utilize any TAC device that they feel is most appropriate at the time of the first and all subsequent dressing changes.

### Data collection

#### Peritoneal fluid and blood

On study day 1, just before application of the allocated TAC dressing, 4 mL of peritoneal fluid and 16 mL of blood will be collected. Peritoneal fluid will be taken directly from the peritoneal cavity and then transferred into sodium-heparin-containing vacutainer tubes (Benton Dickinson Biosciences, Oakville, ON, Canada). Blood will be collected from the patient’s indwelling intravascular catheter or via venipuncture into sodium-heparin containing and clot-activating (serum collection) vacutainer tubes (Benton Dickson Biosciences, Oakville, ON, Canada). Sixteen mL of blood will also be drawn on study days 2, 3, and 7, as well as on study day 28 or at hospital discharge (whichever comes first). Whenever possible, 4 mL of peritoneal fluid will also be removed at these times from the tubing set or Jackson-Pratt drainage tubing among patients fitted with an ABThera™ or Barker’s vacuum pack, respectively. Immediately after collection, blood and peritoneal fluid samples will be gently inverted several times and then immediately spun at 1,200 g for 15 min at 4°C in a swinging bucket centrifuge to remove cellular debris. The cell-free supernatants will subsequently be collected, aliquoted into labeled cryopreservation tubes, and frozen at −80°C until laboratory analyses can be performed.

#### Clinical data

Clinical data will be prospectively recorded at the time of enrollment and until study day 28, hospital discharge, or death. We will collect data on baseline patient demographics; past history; indication for damage control laparotomy; injury or illness severity scores (Acute Physiology and Chronic Health Evaluation-II [APACHE-II] [63]; Sequential Organ Failure Assessment [SOFA] [64], and Injury Severity Score [ISS] [65,66]); fluid balance; need for renal replacement therapy and the Risk, Injury, Failure, Loss, and End-stage-kidney-disease (ESKD) (RIFLE) criteria for acute renal dysfunction [67]; and patient physiology (arterial pH, base deficit, and lactate levels; oxygenation indices and partial pressure of arterial oxygen [PaO_2_]/fraction of inspired oxygen [FIO_2_] ratios; and gastric residual volumes). IAP will also be measured via an indwelling bladder catheter as recommended by the World Society of the Abdominal Compartment Syndrome (WSACS) [68] every 6 h while the abdomen is open and every 12 h once it has been closed.

### Laboratory analyses

We will quantify the peritoneal and plasma concentrations of 65 different mediators relevant to the inflammatory response (Table 2). This broad-based approach will be taken in order to minimize bias with regards to which mediators are measured and to gain a better understanding of which inflammatory pathways are activated after damage control laparotomy and intra-abdominal sepsis or injury.

**Table 2:** Groups of mediators to be measured using Luminex® bead-based multiplexing technology

**Bio-Plex Pro™ Human Cytokine 21-plex Assay (Bio-Rad Laboratories)**	**Bio-Plex Pro™ Human Cytokine 27-plex Assay (Bio-Rad Laboratories)**	**Bio-Plex Pro™ Human Acute Phase 5- + 4-Plex Panel Complete (Bio-Rad Laboratories)**	**Bio-Plex Pro™ Human Custom 8-Plex (Bio-Rad Laboratories)**
IL-1α	IL-1β	Ferritin	IL-17F
IL-2Rα	IL-1ra	Fibrinogen	IL-21
IL-3	IL-2	Procalcitonin	IL-22
IL-12 (p40)	IL-4	Serum amyloid A	IL-23
IL-16	IL-5	tPA	IL-25
IL-18	IL-6	α_2_-macroglobulin	IL-31
CTACK (CCL27)	IL-7	CRP	IL-33
GRO-α (CXCL1)	IL-8	Haptoglobulin	sCD-40L
HGF	IL-9	Serum amyloid P	
IFN-α2	IL-10		
LIF	IL-12 (p70)		
MCP-3 (CCL7)	IL-13		
M-CSF	IL-15		
MIF	IL-17		
MIG (CXCL9)	Basic FGF		
β-NGF	Eotaxin (CCL11)		
SCF	G-CSF		
SCGF-β	GM-CSF		
SDF-1α	IFN-γ		
TNF-β	IP-10 (CXCL10)		
TRAIL	MCP-1 (CCL2)		
	MIP-1α (CCL3)		
	MIP-1β (CCL4)		
	PDGF-BB		
	RANTES (CCL5)		
	TNF-α		
	VEGF		

Basic FGF, Basic fibroblast growth factor; β-NGF, Beta-nerve growth factor; CCL2, Chemokine (C-C motif) ligand 2; CCL3, Chemokine (C-C motif) ligand 3; CCL4, Chemokine (C-C motif) ligand 4; CCL7, Chemokine (C-C motif) ligand 7; CCL11, Chemokine (C-C motif) ligand 11; CCL27, Chemokine (C-C motif) ligand 27; CRP, C-reactive protein; CTACK, Cutaneous T-cell-attracting chemokine; CXCL1, Chemokine (C-X-C motif) ligand 1; CXCL9, Chemokine (C-X-C motif) ligand 9; CXCL10, Chemokine (C-X-C motif) ligand 10; eotaxin, Eosinophil chemotactic protein; G-CSF, Granulocyte colony-stimulating factor; GM-CSF, Granulocyte macrophage colony-stimulating factor; GRO-α, Growth-related oncogene-alpha; HGF, Hepatocyte growth factor; IFN-α2, Interferon alpha-2; IFN-γ, Interferon-gamma; IL-1α, Interleukin-1-alpha; IL-1β, Interleukin-1-beta; IL-1ra, Interleukin-1 receptor antagonist; IL-2, Interleukin-2; IL-2Rα, Interleukin-2 receptor-alpha; IL-3, Interleulin-3; IL-4, Interleukin-4; IL-5, Interleukin-5; IL-6, Interleukin-6; IL-7, Interleukin-7; IL-8, Interleukin-8; IL-9, Interleukin-9; IL-10, Interleukin-10; IL-12 (p40), Interleukin-12 beta subunit; IL-12 (p70), Interleukin-12 (active heterodimer of IL-12 (p40) and IL-12 (p35)); IL-13, Interleukin-13; IL-15, Interleukin-15; IL-16, Interleukin-16; IL-17, Interleukin-17; IL-17F, Interleukin-17F; IL-18, Interleukin-18; IL-21, Interleukin-21; IL-22, Interleukin-22; IL-23, Interleukin-23; IL-25, Interleukin-25; IL-31, Interleukin-31; IL-33, Interleukin-33; IP-10, Interferon gamma-induced protein 10; LIF, Leukemia inhibitory factor; M-CSF, Macrophage colony-stimulating factor; MCP-1, Monocyte chemoattractant protein-1; MCP-3, Monocyte-specific chemokine 3; MIF, Macrophage migration inhibitory factor; MIG, Monokine induced by gamma interferon; MIP-1α, Macrophage inflammatory protein-1-alpha; MIP-1β, Macrophage inflammatory protein-1-beta; PDGF-BB, Platelet-derived growth factor-B homodimer; RANTES, Regulated and normal T cell expressed and secreted; sCD-40L, Soluble cluster of differentiation 40 ligand; SCF, Stem cell factor; SCGF-β, Stem cell growth factor-beta; SDF-1α, Stromal cell-derived factor-1; TNF-α, Tumor necrosis factor-alpha; TNF-β, Tumor necrosis factor-beta; tPA, Tissue plasminogen activator; TRAIL, Tumor necrosis factor-related apoptosis-inducing ligand; VEGF, Vascular endothelial growth factor.

The levels of the mediators listed in Table 2 will be determined using Luminex® technology (EMD Millipore) by an investigator blinded to TAC dressing allocation status. All samples will be screened using a unique combination of the following multiplex kits: Bio-Plex Pro™ Human Cytokine 21-plex Assay, Bio-Plex Pro™ Human Cytokine 27-plex Assay, and Bio-Plex Pro™ Human Acute Phase 5 + 4-plex Panel Complete (Bio-Rad Laboratories). In addition, a Bio-Plex Pro™ Human Custom 8-Plex (IL-17F, -21, -22, -23, -25, -31, -33, and sCD-40L) (Bio-Rad Laboratories) will also be used to provide broadened coverage of Th17 cytokines.

Luminex® combines the sensitivity, specificity, and reproducibility of the Enzyme-Linked ImmunoSorbent Assay (ELISA) with the multiplexing capacity of fluorescent bead immunoassays, thus allowing multiple biomarkers to be measured simultaneously. Using this approach, we will be able to measure, in a cost- and time-efficient manner, a host of analytes using <200 μL of sample (as compared to an ELISA-based approach, which would require >5 mL of sample), while minimizing inter-assay variability. This technology, although new, has been thoroughly tested and validated using human samples [69]. The biomarkers listed in Table 2 will be grouped into four panels based on typical sample dilution and assay buffer compatibilities. Standard curves will then be generated and the data analyzed using Bio-Plex Manager Software version 6.0 (Bio-Rad Laboratories).

### Study endpoints

#### Primary endpoints

As the pro-inflammatory cytokine IL-6 is consistently upregulated following intra-abdominal sepsis or injury and has been reported to correlate with outcomes (Table 2), the primary endpoint is the difference in the plasma concentration of IL-6 at 24 and 48 h after dressing application between patients randomized to TAC with the ABThera™ *versus* Barker’s vacuum pack after damage control laparotomy.

#### Secondary endpoints and exploration of clinical efficacy and safety

Secondary endpoints of interest include trial feasibility (number of patients enrolled/number of eligible candidates) and the differential effects of the allocated TAC dressings on several physiological and organ dysfunction outcomes, including: (1) the plasma concentration of TNF-α and IL-1, -8, -10, and −12 at 24 and 48 h; (2) the collective peritoneal and systemic mediator profiles (see below); (3) the activation potential of peritoneal fluid [70,71]; (4) peritoneal fluid drainage volume; (5) postoperative daily fluid balance; (6) SOFA scores and individual organ system components of these scores; (7) the PaO_2_/FIO_2_ ratio; (8) vasopressor requirements and arterial lactate levels; (9) need for renal replacement therapy; (10) RIFLE criteria; (11) APACHE-II scores; and (12) 24 h enteral tolerance (if no gastrointestinal anastomosis was performed).

Secondary clinical efficacy and safety endpoints of interest include: (1) in-hospital death; (2) fascial closure rate and days with fascial closure for the month after admission to hospital; (3) ventilator-free days for the month after admission to hospital; (4) ICU-free days from the month after admission to hospital; (5) hospital-free days from the month after admission to hospital; and (6) risk of renal replacement therapy and days free of renal replacement therapy from the month after admission to hospital. Further, as some have suggested that negative pressure peritoneal therapy may be associated with an increased risk of abdominal fistula formation or development of intra-abdominal hypertension/recurrent abdominal compartment syndrome [72-74], additional safety outcomes include: (1) intestinal and enteroatmospheric fistula formation; (2) daily IAP; (3) daily WSACS intra-abdominal hypertension grading classification; and (4) risk of abdominal compartment syndrome development.

### Sample size

No clinical data yet exist on the effect of negative pressure peritoneal therapy on the inflammatory response after damage control laparotomy for intra-abdominal sepsis or injury. Thus, estimates are not available to allow accurate sample size estimation. We therefore propose to undertake a study among a convenience sample of 46 adults. As there are approximately 40 to 50 patients who undergo damage control laparotomy and open abdominal management per year at our institution, this trial should be able to be completed within a 2-year time period.

### Statistical analyses

All continuous variables will be summarized using histograms and measures of central tendency to determine their underlying distribution before statistical descriptions or analyses are conducted. Means (with standard deviations) and medians (with interquartile ranges) will be used to summarize normal and skewed data, respectively. Non-correlated means and medians will be compared using t-tests and Wilcoxon rank sum tests while dependent means and medians will be compared using paired t-tests and Wilcoxon signed rank tests. Differences in proportions will be compared using Fisher’s exact test and risk ratios with associated 95% confidence intervals.

For the analysis of the primary endpoint, we will use a mixed-effects repeated measures linear model to compare the effects of the ABThera™ and Barker’s vacuum pack on changes in plasma IL-6 concentration at 24 and 48 h after TAC dressing application [75,76]. For longitudinal studies, mixed-effects models have previously been shown to be superior to conventional last-observation-carried-forward approaches for handling missed observations during follow-up [77,78]. This approach will also be used to compare the plasma concentrations of TNF-α and IL-1, -8, -10, and −12 between groups at 24 and 48 h in the analysis of secondary endpoints.

Multidimensional analysis will be used to assess whether the allocated TAC dressing has an effect on the collective peritoneal and plasma mediator profiles of the study patients. Principal components analysis will be used to reduce the mediator concentration variables into a smaller set of components that account for most of the variability in the data. Component scores will subsequently be calculated along the reduced number dimensions, after which we will attempt to distinguish between patients treated with the ABThera™ and Barker’s vacuum pack using discriminant or latent class analysis. A similar approach will be performed in order to relate inflammatory mediator concentrations to outcomes.

Results will be stratified according to the primary indication for damage control laparotomy (intra-abdominal sepsis or abdominal trauma). While clinical efficacy and safety data will be analyzed according to intention-to-treat methods, mediator data will be examined using per-protocol techniques, with per-protocol treatment being defined as the allocated TAC dressing having been in place for at least 24 h. As the number of conducted statistical tests will be large, we will use the false discovery rate (FDR) procedure developed by Benjamini and Hochberg to restrict the proportion of incorrectly rejected null hypotheses to 0.05 [79-81]. All tests will be two-sided, and only those with an FDR-corrected *P* value (that is q-value) <0.05 will be considered statistically significant [79]. Stata version 12.0 (Stata Corp., College Station, TX, USA) will be used for all analyses.

### Ethics approval, trial registration, and role of the sponsor

The study protocol has been approved by the Conjoint Health Research Ethics Board (CHREB) at the University of Calgary, and is registered online at ClinicalTrials.gov (identifier NCT01355094). Costs of the study will be covered by an investigator-initiated trial funding agreement between the principal investigator (AWK), KCI USA (the manufacturer of the ABThera™ Open Abdomen NPT System), and the Governors of the University of Calgary (KCI contract number: KCI Clinical/UniversityCalgaryAlbertaHealth/082611-000/7). KCI USA has had no role in the design or conduct of the study and will have no role in the collection, management, analysis, or interpretation of the data or preparation, review, or approval of the final manuscript.

## Discussion

This will be the first study to compare the systemic inflammatory response among critically ill or injured adults fitted with a TAC that may provide active negative pressure peritoneal therapy with a commonly used TAC technique that provides potentially less efficient peritoneal negative pressure, the Barker’s vacuum pack [10]. This study aims to afford a robust prospective description of the peritoneal and systemic inflammatory response after damage control laparotomy for intra-abdominal sepsis or injury. A secondary aim is to gather necessary pilot information related to trial feasibility, intervention safety, and design deficiencies needed to inform construction of a multicenter RCT comparing clinical outcomes among patients fitted with the ABThera™ *versus* Barker’s vacuum pack.

In this trial, study patients in both treatment arms will undergo planned re-operation with attempts at abdominal fascial closure approximately 24 to 72 h after damage control laparotomy. Although one RCT (the RELAP trial) reported that planned re-laparatomy after an initial emergency laparotomy for secondary peritonitis resulted in increased healthcare utilization and costs as compared to re-laparotomy on demand [82,83], these findings likely cannot be generalized to our study’s source population. In the RELAP trial, the investigators excluded patients that were managed with temporary operative techniques such as intra-abdominal gauze packing and stapled intestinal resections without reanastomosis [82]. Moreover, 89% of the patients allocated to planned re-laparotomy received intraoperative primary abdominal fascial closure [82]. Thus, very few, if any, of the patients in the planned re-laparotomy group likely received damage control laparotomy.

Several difficulties were encountered in designing the Intra-peritoneal Vacuum trial. The trial is demanding for study investigators as damage control laparotomy must be performed emergently and is often done at night in our center when research coordinators and laboratory members are unavailable. For these reasons, the randomization interface and enrollment process were simplified in order to prevent delays in application of the TAC device, which could alter patient outcomes. Further, as no objective, evidence-based consensus indications for damage control laparotomy or open abdominal management have yet been described, we were forced to utilize a pragmatic study design, which attempts to mimic actual surgical practice. However, in order to increase understanding, we planned *a priori* to conduct a nested prospective cohort study that will explore indications used for open abdominal management among attending trauma and general surgeons at our center.

As compared to previous translational human studies of physical therapies to reduce peritoneal and systemic inflammation among critically ill adults, this trial will utilize novel laboratory analyses and rigorous statistical techniques. In order to avoid bias with regards to which mediators are measured, we will use the validated Luminex® multiplexing technology to assay a host of pro-inflammatory mediators simultaneously in a cost- and time-efficient manner, maximizing use of the obtained patient samples [69]. Further, although many authors have conducted comparisons of cytokine concentrations with study endpoints, they frequently ignore the correlation between mediators measured within individual patients over time. Thus, when examining the relationship between cytokines and TAC method, we will use validated statistical techniques that estimate and incorporate this correlation, and which appropriately handle missed observations during follow-up. Finally, in order to collectively analyze the difference in multiple measured mediators between TAC treatment groups, we will also use multidimensional analyses, including principal components analysis.

This trial has limitations. Although we will be combining the inflammatory mediator concentrations and outcome data for patients with both intra-abdominal sepsis and injury, which may be different, we will stratify our analyses to examine if differences exist between patient diagnoses. Moreover, as the primary objective of this trial is to explore the influence of active abdominal therapy on the systemic inflammatory response among critically ill or injured adults, we will principally use a per-protocol method of analysis. As this method of analysis may not be appropriate or as clinically useful for the exploration of safety and efficacy, we will also utilize an intention-to-treat method of analysis when comparing these endpoints. Importantly, however, as we were unable to estimate the required sample size, we may be underpowered to detect differences in the plasma pro-inflammatory mediator concentrations (or clinical and safety endpoints) among patients randomized to TAC with the ABThera™ *versus* Barker’s vacuum pack.

In conclusion, the Intra-peritoneal Vacuum Trial will be a single-center RCT. Results from this study will lead to an improved understanding of the effect of active negative pressure peritoneal therapy on the systemic inflammatory response to intra-abdominal sepsis or injury after damage control laparotomy. The study will also gather pilot information needed to inform creation of a future multicenter RCT comparing clinical outcomes among those undergoing TAC with the ABThera™ *versus* the Barker’s vacuum pack after damage control laparotomy.

## Trial status

The first patient was enrolled into the Intra-peritoneal Vacuum Trial on 29 September 2011, and we are still actively recruiting patients. At the time of writing this manuscript, 37 patients had been enrolled into the trial, the majority of which have already given delayed informed consent for use of their data. Final results of the study are expected to be prepared for publication near the conclusion of 2013.

## Abbreviations

APACHE-II: Acute physiology and chronic health evaluation-II; ELISA: Enzyme-linked ImmunoSorbent assay; ESKD: End stage kidney disease; FIO2: Fraction of inspired oxygen; FMC: Foothills medical center; IAP: Intra-abdominal pressure; KCI: Kinetic Concepts Inc; ICU: Intensive care unit; ISS: Injury severity score; PaO2: Partial pressure of arterial oxygen; RCT: Randomized controlled trial; RIFLE: Risk injury, failure, loss, and end-stage-kidney-disease; SOFA: Sequential organ failure assessment; TAC: Temporary abdominal closure; WSACS: World Society of the Abdominal Compartment Syndrome

## Competing interests

AWK has an investigator-initiated trial funding agreement with Kinetic Concepts Incorporated (KCI) USA and the Governors of the University of Calgary for the clinical and laboratory costs of conducting the study (KCI contract number: KCI Clinical/UniversityCalgaryAlbertaHealth/082611-000/7). KCI USA will have no role in the design or conduct of the study; collection, management, analysis, or interpretation of the data; or preparation, review, or approval of the manuscript. The remaining authors have no competing interests to declare.

## Authors’ contributions

AWK conceived the study. CGB, PBM, CT, CJD, and AWK designed the inclusion/exclusion criteria, specified the interventions, developed the randomization scheme, and submitted the protocol for ethics approval. DJR, CNJ, CT, CJD, CS, SGR, PK, and AWK developed and refined the sample collection procedures. CNJ, CL, PK, and AWK developed the laboratory analyses plan and all authors selected the mediators for measurement. CT and JX developed the trial website while CT developed the online randomization tool. All authors selected the primary and secondary endpoints of interest. DJR and PDF designed the statistical analysis plan. DJR, CNJ, CGB, CT, JX, CJD, CS, SGR, PK, and AWK assist in patient enrollment and revised protocol drafts and data collection forms. DJR, CNJ, CT, JX, CS, SGR, and AWK provide trial oversight on trial coordination and implementation. DJR and CNJ drafted the protocol for publication. All authors afforded critical input on the manuscript and saw and approved the final version before submission for publication.

## Authors’ information

DJR is a surgery and Clinician Investigator Program resident who is presently conducting a Doctor of Philosophy in epidemiology with a thesis on damage control surgery at the University of Calgary. CGB and AWK are academic trauma and acute care surgeons while AWK is also an intensivist at the Foothills Medical Center. AWK is also the past President of the Trauma Association of Canada and a member of the Executive Committee of the World Society of the Abdominal Compartment Syndrome. PDF is a biostatistician with experience in randomized trial design and analysis of complex correlated data sets. CNJ, CL, and PK are basic scientists with an interest in inflammation and infection while PK is the Director of the Calvin, Phoebe and Joan Snyder Institute of Chronic Diseases, which is renowned for its study of sepsis and infection. PK is also the founding member of the Alberta Sepsis Network, an Alberta Innovates - Health Solutions-funded team grant focusing on the development of novel science and technology, including the conduct of randomized controlled trials, for the understanding and treatment of sepsis.

## References

[B1] Murray CJ Lopez AD Mortality by cause for eight regions of the world: global burden of disease study Lancet 1997 349 1269 1276 10.1016/S0140-6736(96)07493-4 9142060

[B2] Chow AW Evans GA Nathens AB Ball CG Hansen G Harding GK Kirkpatrick AW Weiss K Zhanel GG Canadian practice guidelines for surgical intra-abdominal infections Can J Infect Dis Med Microbiol 2010 21 11 37 21358883 10.1155/2010/580340 PMC2852280

[B3] Stelfox HT Bobranska-Artiuch B Nathens A Straus SE Quality indicators for evaluating trauma care: a scoping review Arch Surg 2010 145 286 295 10.1001/archsurg.2009.289 20231631

[B4] Nishijima DK Simel DL Wisner DH Holmes JF Does this adult patient have a blunt intra-abdominal injury? JAMA 2012 307 1517 1527 10.1001/jama.2012.422 22496266 PMC4966670

[B5] Chovanes J Cannon JW Nunez TC The evolution of damage control surgery Surg Clin North Am 2012 92 859 875 vii-viii 10.1016/j.suc.2012.04.002 22850151

[B6] Waibel BH Rotondo MF Damage control for intra-abdominal sepsis Surg Clin North Am 2012 92 243 257 vii 10.1016/j.suc.2012.01.006 22414411

[B7] Stone HH Strom PR Mullins RJ Management of the major coagulopathy with onset during laparotomy Ann Surg 1983 197 532 535 10.1097/00000658-198305000-00005 6847272 PMC1353025

[B8] Burch JM Ortiz VB Richardson RJ Martin RR Mattox KL Jordan GL Jr Abbreviated laparotomy and planned reoperation for critically injured patients Ann Surg 1992 215 476 483 discussion 483–484 10.1097/00000658-199205000-00010 1616384 PMC1242479

[B9] Rotondo MF Schwab CW McGonigal MD Phillips GR III Fruchterman TM Kauder DR Latenser BA Angood PA ‘Damage control’: an approach for improved survival in exsanguinating penetrating abdominal injury J Trauma 1993 35 375 382 discussion 382–383 10.1097/00005373-199309000-00008 8371295

[B10] Roberts DJ Zygun DA Grendar J Ball CG Robertson HL Ouellet JF Cheatham ML Kirkpatrick AW Negative-pressure wound therapy for critically ill adults with open abdominal wounds: a systematic review J Trauma Acute Care Surg 2012 73 629 639 10.1097/TA.0b013e31825c130e 22929494

[B11] Holzheimer RG Schein M Wittmann DH Inflammatory response in peritoneal exudate and plasma of patients undergoing planned relaparotomy for severe secondary peritonitis Arch Surg 1995 130 1314 1319 discussion 1319–1320 10.1001/archsurg.1995.01430120068010 7492280

[B12] Scheingraber S Bauerfeind F Bohme J Dralle H Limits of peritoneal cytokine measurements during abdominal lavage treatment for intraabdominal sepsis Am J Surg 2001 181 301 308 10.1016/S0002-9610(01)00587-6 11438263

[B13] Mayberry JC Welker KJ Goldman RK Mullins RJ Mechanism of acute ascites formation after trauma resuscitation Arch Surg 2003 138 773 776 10.1001/archsurg.138.7.773 12860760

[B14] Fink MP Delude RL Epithelial barrier dysfunction: a unifying theme to explain the pathogenesis of multiple organ dysfunction at the cellular level Crit Care Clin 2005 21 177 196 10.1016/j.ccc.2005.01.005 15781156

[B15] Ince C The microcirculation is the motor of sepsis Crit Care 2005 Suppl 4 S13 S19 16168069 10.1186/cc3753 PMC3226164

[B16] van Veen SQ Meijers JC Levi M van Gulik TM Boermeester MA Effects of intra-abdominal administration of recombinant tissue plasminogen activator on coagulation, fibrinolysis and inflammatory responses in experimental polymicrobial peritonitis Shock 2007 27 534 541 10.1097/01.shk.0000246897.27574.1b 17438459

[B17] el Zakaria R Li N Garrison RN Mechanisms of direct peritoneal resuscitation-mediated splanchnic hyperperfusion following hemorrhagic shock Shock 2007 27 436 442 10.1097/01.shk.0000245017.86117.4e 17414428 PMC2121218

[B18] Kubiak BD Albert SP Gatto LA Snyder KP Maier KG Vieau CJ Roy S Nieman GF Peritoneal negative pressure therapy prevents multiple organ injury in a chronic porcine sepsis and ischemia/reperfusion model Shock 2010 34 525 534 10.1097/SHK.0b013e3181e14cd2 20823698

[B19] Hendriks T Bleichrodt RP Lomme RM De Man BM van Goor H Buyne OR Peritoneal cytokines predict mortality after surgical treatment of secondary peritonitis in the rat J Am Coll Surg 2010 211 263 270 10.1016/j.jamcollsurg.2010.03.038 20670866

[B20] Ravishankaran P Shah AM Bhat R Correlation of interleukin-6, serum lactate, and C-reactive protein to inflammation, complication, and outcome during the surgical course of patients with acute abdomen J Interferon Cytokine Res 2011 31 685 690 10.1089/jir.2011.0021 21923250

[B21] Li P Xu Q Ji Z Gao Y Zhang X Duan Y Guo Z Zheng B Guo X Wu X Comparison of surgical stress between laparoscopic and open appendectomy in children J Pediatr Surg 2005 40 1279 1283 10.1016/j.jpedsurg.2005.05.011 16080932

[B22] Roelofsen H Alvarez-Llamas G Dijkstra M Breitling R Havenga K Bijzet J Zandbergen W de Vries MP Ploeg RJ Vonk RJ Analyses of intricate kinetics of the serum proteome during and after colon surgery by protein expression time series Proteomics 2007 7 3219 3228 10.1002/pmic.200601047 17806085

[B23] Xiao W Mindrinos MN Seok J Cuschieri J Cuenca AG Gao H Hayden DL Hennessy L Moore EE Minei JP Bankey PE Johnson JL Sperry J Nathens AB Billiar TR West MA Brownstein BH Mason PH Baker HV Finnerty CC Jeschke MG Lopez MC Klein MB Gamelli RL Gibran NS Arnoldo B Xu W Zhang Y Calvano SE McDonald-Smith GP Inflammation and Host Response to Injury Large-Scale Collaborative Research Program, et al A genomic storm in critically injured humans J Exp Med 2011 208 2581 2590 10.1084/jem.20111354 22110166 PMC3244029

[B24] Liu T Qian WJ Gritsenko MA Xiao W Moldawer LL Kaushal A Monroe ME Varnum SM Moore RJ Purvine SO Maier RV Davis RW Tompkins RG Camp DG III Smith RD Inflammation and the Host Response to Injury Large Scale Collaborative Research Programm High dynamic range characterization of the trauma patient plasma proteome Mol Cell Proteomics 2006 5 1899 1913 10.1074/mcp.M600068-MCP200 16684767 PMC1783978

[B25] Hack CE De Groot ER Felt-Bersma RJ Nuijens JH Strack Van Schijndel RJ Eerenberg-Belmer AJ Thijs LG Aarden LA Increased plasma levels of interleukin-6 in sepsis Blood 1989 74 1704 1710 2790194

[B26] Latifi SQ O’Riordan MA Levine AD Stallion A Persistent elevation of serum interleukin-6 in intraabdominal sepsis identifies those with prolonged length of stay J Pediatr Surg 2004 39 1548 1552 10.1016/j.jpedsurg.2004.06.015 15486902

[B27] Gletsu N Lin E Zhu JL Khaitan L Ramshaw BJ Farmer PK Ziegler TR Papanicolaou DA Smith CD Increased plasma interleukin 6 concentrations and exaggerated adipose tissue interleukin 6 content in severely obese patients after operative trauma Surgery 2006 140 50 57 10.1016/j.surg.2006.01.018 16857442

[B28] Frangen TM Bogdanski D Schinkel C Roetman B Kalicke T Muhr G Koller M Systemic IL-17 after severe injuries Shock 2008 29 462 467 17909455 10.1097/shk.0b013e3181598a9d

[B29] Zeilhofer HU Schorr W Role of interleukin-8 in neutrophil signaling Curr Opin Hematol 2000 7 178 182 10.1097/00062752-200005000-00009 10786656

[B30] Bingold TM Ziesche E Scheller B Sadik CD Franck K Just L Sartorius S Wahrmann M Wissing H Zwissler B Pfeilschifter J Muhl H Interleukin-22 detected in patients with abdominal sepsis Shock 2010 34 337 340 10.1097/SHK.0b013e3181dc07b1 20220564

[B31] Rongione AJ Kusske AM Ashley SW Reber HA McFadden DW Interleukin-10 prevents early cytokine release in severe intraabdominal infection and sepsis J Surg Res 1997 70 107 112 10.1006/jsre.1997.5071 9237883

[B32] van Berge Henegouwen MI van der Poll T van Deventer SJ Gouma DJ Peritoneal cytokine release after elective gastrointestinal surgery and postoperative complications Am J Surg 1998 175 311 316 10.1016/S0002-9610(98)00010-5 9568659

[B33] Kimura A Ono S Hiraki S Takahata R Tsujimoto H Miyazaki H Kinoshita M Hatsuse K Saitoh D Hase K Yamamoto J The postoperative serum interleukin-15 concentration correlates with organ dysfunction and the prognosis of septic patients following emergency gastrointestinal surgery J Surg Res 2012 175 e83 e88 10.1016/j.jss.2011.12.003 22341349

[B34] Pappu R Ramirez-Carrozzi V Sambandam A The interleukin-17 cytokine family: critical players in host defence and inflammatory diseases Immunology 2011 134 8 16 10.1111/j.1365-2567.2011.03465.x 21726218 PMC3173690

[B35] Freitas A Alves-Filho JC Victoni T Secher T Lemos HP Sonego F Cunha FQ Ryffel B IL-17 receptor signaling is required to control polymicrobial sepsis J Immunol 2009 182 7846 7854 10.4049/jimmunol.0803039 19494309

[B36] Alves-Filho JC Sonego F Souto FO Freitas A Verri WA Jr Auxiliadora-Martins M Basile-Filho A McKenzie AN Xu D Cunha FQ Liew FY Interleukin-33 attenuates sepsis by enhancing neutrophil influx to the site of infection Nat Med 2010 16 708 712 10.1038/nm.2156 20473304

[B37] Dib M Zhao X Wang X Andersson E Drewsen G Andersson R Acute phase response in acute pancreatitis: a comparison with abdominal sepsis Scand J Gastroenterol 2003 38 1072 1077 10.1080/00365520310005442 14621283

[B38] Brenner T Hofer S Rosenhagen C Steppan J Lichtenstern C Weitz J Bruckner T Lukic IK Martin E Bierhaus A Hoffmann U Weigand MA Macrophage migration inhibitory factor (MIF) and manganese superoxide dismutase (MnSOD) as early predictors for survival in patients with severe sepsis or septic shock J Surg Res 2010 164 e163 e171 10.1016/j.jss.2010.05.004 20863520

[B39] Calandra T Echtenacher B Roy DL Pugin J Metz CN Hultner L Heumann D Mannel D Bucala R Glauser MP Protection from septic shock by neutralization of macrophage migration inhibitory factor Nat Med 2000 6 164 170 10.1038/72262 10655104

[B40] Reith HB Mittelkotter U Wagner R Thiede A Procalcitonin (PCT) in patients with abdominal sepsis Intensive Care Med 2000 Suppl 2 S165 S169 18470713 10.1007/BF02900731

[B41] Cziupka K Busemann A Partecke LI Potschke C Rath M Traeger T Koerner P von Bernstorff W Kessler W Diedrich S Weiss FU Maier S Broker BM Heidecke CD Tumor necrosis factor-related apoptosis-inducing ligand (TRAIL) improves the innate immune response and enhances survival in murine polymicrobial sepsis Crit Care Med 2010 38 2169 2174 10.1097/CCM.0b013e3181eedaa8 20657274

[B42] Wang S TRAIL: a sword for killing tumors Curr Med Chem 2010 17 3309 3317 10.2174/092986710793176285 20712573

[B43] McBeth PB Leger C Ball CG Ouellet JF Tiruta C Laupland KB Kubes P Roberts DJ Shahpori R Kirkpatrick AW Intra-abdominal hypertension and intra-abdominal sepsis: critical concepts and possibilities Int J Intensive Care 2011 18 10 19

[B44] Casey LC Balk RA Bone RC Plasma cytokine and endotoxin levels correlate with survival in patients with the sepsis syndrome Ann Intern Med 1993 119 771 778 10.7326/0003-4819-119-8-199310150-00001 8379598

[B45] Roumen RM Hendriks T van der Ven-Jongekrijg J Nieuwenhuijzen GA Sauerwein RW van der Meer JW Goris RJ Cytokine patterns in patients after major vascular surgery, hemorrhagic shock, and severe blunt trauma. Relation with subsequent adult respiratory distress syndrome and multiple organ failure Ann Surg 1993 218 769 776 10.1097/00000658-199312000-00011 8257227 PMC1243073

[B46] Pinsky MR Vincent JL Deviere J Alegre M Kahn RJ Dupont E Serum cytokine levels in human septic shock. Relation to multiple-system organ failure and mortality Chest 1993 103 565 575 10.1378/chest.103.2.565 8432155

[B47] Svoboda P Kantorova I Ochmann J Dynamics of interleukin 1, 2, and 6 and tumor necrosis factor alpha in multiple trauma patients J Trauma 1994 36 336 340 10.1097/00005373-199403000-00009 8145312

[B48] Pettila V Hynninen M Takkunen O Kuusela P Valtonen M Predictive value of procalcitonin and interleukin 6 in critically ill patients with suspected sepsis Intensive Care Med 2002 28 1220 1225 10.1007/s00134-002-1416-1 12209268

[B49] Spindler-Vesel A Wraber B Vovk I Kompan L Intestinal permeability and cytokine inflammatory response in multiply injured patients J Interferon Cytokine Res 2006 26 771 776 10.1089/jir.2006.26.771 17032171

[B50] Jawa RS Anillo S Huntoon K Baumann H Kulaylat M Interleukin-6 in surgery, trauma, and critical care part II: clinical implications J Intensive Care Med 2011 26 73 87 21464062 10.1177/0885066610384188 PMC6223019

[B51] Quyn AJ Johnston C Hall D Chambers A Arapova N Ogston S Amin AI The open abdomen and temporary abdominal closure systems–historical evolution and systematic review Colorectal Dis 2012 14 e429 e438 10.1111/j.1463-1318.2012.03045.x 22487141

[B52] Bee TK Croce MA Magnotti LJ Zarzaur BL Maish GO III Minard G Schroeppel TJ Fabian TC Temporary abdominal closure techniques: a prospective randomized trial comparing polyglactin 910 mesh and vacuum-assisted closure J Trauma 2008 65 337 342 discussion 342–344 10.1097/TA.0b013e31817fa451 18695468

[B53] Clinical guidelines for the management of the open abdomen with ABThera™ Open Abdomen Negative Pressure Therapy System for active abdominal therapy [http://www.peritonealvac.com/ABThera%20Clinical%20Guidelines.pdf]

[B54] Brock WB Barker DE Burns RP Temporary closure of open abdominal wounds: the vacuum pack Am Surg 1995 61 30 35 7832378

[B55] Smith LA Barker DE Chase CW Somberg LB Brock WB Burns RP Vacuum pack technique of temporary abdominal closure: a four-year experience Am Surg 1997 63 1102 1107 discussion 1107–1108 9393260

[B56] Barker DE Kaufman HJ Smith LA Ciraulo DL Richart CL Burns RP Vacuum pack technique of temporary abdominal closure: a 7-year experience with 112 patients J Trauma 2000 48 201 206 discussion 206–207 10.1097/00005373-200002000-00001 10697075

[B57] Schulz KF Altman DG Moher D CONSORT Group CONSORT 2010 Statement: updated guidelines for reporting parallel group randomised trials Trials 2010 11 32 10.1186/1745-6215-11-32 20334632 PMC2857832

[B58] Sammons A Delgado A Cheatham ML In-vitro pressure manifolding distribution evaluation of the ABThera Open Abdomen Negative Pressure Therapy System, V.A.C. Abdominal Derssing System, and Barker’s vacuum-pack technique, conducted under dynamic conditions 2009 San Antonio, TX: [Abst P 078] Clinical Symposium on Advances in Skin & Wound Care

[B59] Diaz JJ Jr Cullinane DC Dutton WD Jerome R Bagdonas R Bilaniuk JW Collier BR Como JJ Cumming J Griffen M Gunter OL Kirby J Lottenburg L Mowery N Riordan WP Jr Martin N Platz J Stassen N Winston ES The management of the open abdomen in trauma and emergency general surgery: part 1-damage control J Trauma 2010 68 1425 1438 10.1097/TA.0b013e3181da0da5 20539186

[B60] Asensio JA Petrone P O’Shanahan G Kuncir EJ Managing exsanguination: what we know about damage control/bailout is not enough Proc (Bayl Univ Med Cent) 2003 16 294 296 16278701 10.1080/08998280.2003.11927917 PMC1200784

[B61] Ivatury RR Malhotra AK Wilson WC, Grande CM, Hoyt DB Damage Control Trauma: Emergency Resuscitation, Perioperative Anesthesia, Surgical Management 2007 New York, NY: Informa Healthcare USA, Inc 405 416

[B62] Kaplan M Banwell P Orgill DP Ivatury RR Demetriades D Moore FA Miller P Nicholas J Henry S Guidelines for the management of the open abdomen Wounds-Compend Clin Res Pract 2005 Suppl 1 24

[B63] Knaus WA Draper EA Wagner DP Zimmerman JE APACHE II: a severity of disease classification system Crit Care Med 1985 13 818 829 10.1097/00003246-198510000-00009 3928249

[B64] Vincent JL Moreno R Takala J Willatts S De Mendonca A Bruining H Reinhart CK Suter PM Thijs LG The SOFA (sepsis-related organ failure assessment) score to describe organ dysfunction/failure. On behalf of the working group on sepsis-related problems of the European society of intensive care medicine Intensive Care Med 1996 22 707 710 10.1007/BF01709751 8844239

[B65] Baker SP O’Neill B Haddon W Jr Long WB The injury severity score: a method for describing patients with multiple injuries and evaluating emergency care J Trauma 1974 14 187 196 10.1097/00005373-197403000-00001 4814394

[B66] Linn S The injury severity score–importance and uses Ann Epidemiol 1995 5 440 446 10.1016/1047-2797(95)00059-3 8680606

[B67] Bellomo R Ronco C Kellum JA Mehta RL Palevsky P Acute Dialysis Quality Initiative workgroup Acute renal failure - definition, outcome measures, animal models, fluid therapy and information technology needs: the second international consensus conference of the acute dialysis quality initiative (ADQI) group Crit Care 2004 8 R204 R212 10.1186/cc2872 15312219 PMC522841

[B68] Cheatham ML Malbrain ML Kirkpatrick A Sugrue M Parr M De Waele J Balogh Z Leppaniemi A Olvera C Ivatury R D’Amours S Wendon J Hillman K Wilmer A Results from the international conference of experts on intra-abdominal hypertension and abdominal compartment syndrome. II. Recommendations Intensive Care Med 2007 33 951 962 10.1007/s00134-007-0592-4 17377769

[B69] Dupont NC Wang K Wadhwa PD Culhane JF Nelson EL Validation and comparison of luminex multiplex cytokine analysis kits with ELISA: determinations of a panel of nine cytokines in clinical sample culture supernatants J Reprod Immunol 2005 66 175 191 10.1016/j.jri.2005.03.005 16029895 PMC5738327

[B70] Shah SK Jimenez F Walker PA Aroom KR Xue H Feeley TD Uray KS Norbury KC Stewart RH Laine GA Cox CS Jr A novel mechanism for neutrophil priming in trauma: potential role of peritoneal fluid Surgery 2010 148 263 270 10.1016/j.surg.2010.03.019 20466401 PMC2905488

[B71] Shah SK Jimenez F Walker PA Xue H Feeley TD Uray KS Norbury KC Stewart RH Laine GA Cox CS Jr Peritoneal fluid: a potential mechanism of systemic neutrophil priming in experimental intra-abdominal sepsis Am J Surg 2012 203 211 216 10.1016/j.amjsurg.2010.12.012 21679918 PMC3177024

[B72] Ouellet JF Ball CG Recurrent abdominal compartment syndrome induced by high negative pressure abdominal closure dressing J Trauma 2011 71 785 786 21909013 10.1097/TA.0b013e31822bbde5

[B73] Rao M Burke D Finan PJ Sagar PM The use of vacuum-assisted closure of abdominal wounds: a word of caution Colorectal Dis 2007 9 266 268 10.1111/j.1463-1318.2006.01154.x 17298627

[B74] Fischer JE A cautionary note: the use of vacuum-assisted closure systems in the treatment of gastrointestinal cutaneous fistula may be associated with higher mortality from subsequent fistula development Am J Surg 2008 196 1 2 10.1016/j.amjsurg.2008.01.001 18355795

[B75] Ware JH Linear models for the analysis of longitudinal studies Am Stat 1985 39 95 101 10.2307/2682803

[B76] Laird NM Donnelly C Ware JH Longitudinal studies with continuous responses Stat Methods Med Res 1992 1 225 247 10.1177/096228029200100302 1341659

[B77] Liu G Gould AL Comparison of alternative strategies for analysis of longitudinal trials with dropouts J Biopharm Stat 2002 12 207 226 10.1081/BIP-120015744 12413241

[B78] Mallinckrodt CH Clark WS David SR Accounting for dropout bias using mixed-effects models J Biopharm Stat 2001 11 9 21 10.1081/BIP-100104194 11459446

[B79] Benjamini Y Hochberg Y Controlling the false discovery rate: a practical and powerful approach to multiple testing J R Statist Soc B 1995 57 289 300

[B80] Newson R Multiple-test procedures and smile plots Stata J 2003 3 109 132

[B81] Newson RB Frequentist q-values for multiple-test procedures Stata J 2010 10 568 584

[B82] van Ruler O Mahler CW Boer KR Reuland EA Gooszen HG Opmeer BC de Graaf PW Lamme B Gerhards MF Steller EP van Till JW de Borgie CJ Gouma DJ Reitsma JB Boermeester MA Dutch Peritonitis Study Group Comparison of on-demand vs planned relaparotomy strategy in patients with severe peritonitis: a randomized trial JAMA 2007 298 865 872 10.1001/jama.298.8.865 17712070

[B83] Opmeer BC Boer KR van Ruler O Reitsma JB Gooszen HG de Graaf PW Lamme B Gerhards MF Steller EP Mahler CM Obertop H Gouma DJ Bossuyt PM de Borgie CA Boermeester MA Costs of relaparotomy on-demand versus planned relaparotomy in patients with severe peritonitis: an economic evaluation within a randomized controlled trial Crit Care 2010 14 R97 10.1186/cc9032 20507557 PMC2911734

